# Pax6 associates with H3K4-specific histone methyltransferases Mll1, Mll2, and Set1a and regulates H3K4 methylation at promoters and enhancers

**DOI:** 10.1186/s13072-016-0087-z

**Published:** 2016-09-09

**Authors:** Jian Sun, Yilin Zhao, Rebecca McGreal, Yamit Cohen-Tayar, Shira Rockowitz, Carola Wilczek, Ruth Ashery-Padan, David Shechter, Deyou Zheng, Ales Cvekl

**Affiliations:** 1 Department of Genetics, Albert Einstein College of Medicine, Bronx, NY 10461 USA; 2 Department of Ophthalmology and Visual Sciences, Albert Einstein College of Medicine, Bronx, NY 10461 USA; 3 Department of Human Molecular Genetics and Biochemistry, Faculty of Medicine, Sagol school of Neuroscience, Tel-Aviv University, Tel Aviv, 69978 Israel; 4 Department of Biochemistry, Albert Einstein College of Medicine, Bronx, NY 10461 USA; 5 Department of Neurology, Albert Einstein College of Medicine, Bronx, NY 10461 USA; 6 Department of Neuroscience, Albert Einstein College of Medicine, Bronx, NY 10461 USA

**Keywords:** Pax6, Histone methylation, Mll1, Mll2, Set1a, Enhancer, Lens, Retinal pigmented epithelium, Plekha1

## Abstract

**Background:**

Pax6 is a key regulator of the entire cascade of ocular lens formation through specific binding to promoters and enhancers of batteries of target genes. The promoters and enhancers communicate with each other through DNA looping mediated by multiple protein–DNA and protein–protein interactions and are marked by specific combinations of histone posttranslational modifications (PTMs). Enhancers are distinguished from bulk chromatin by specific modifications of core histone H3, including H3K4me1 and H3K27ac, while promoters show increased H3K4me3 PTM. Previous studies have shown the presence of Pax6 in as much as 1/8 of lens-specific enhancers but a much smaller fraction of tissue-specific promoters. Although Pax6 is known to interact with EP300/p300 histone acetyltransferase responsible for generation of H3K27ac, a potential link between Pax6 and histone H3K4 methylation remains to be established.

**Results:**

Here we show that Pax6 co-purifies with H3K4 methyltransferase activity in lens cell nuclear extracts. Proteomic studies show that Pax6 immunoprecipitates with Set1a, Mll1, and Mll2 enzymes, and their associated proteins, i.e., Wdr5, Rbbp5, Ash2l, and Dpy30. ChIP-seq studies using chromatin prepared from mouse lens and cultured lens cells demonstrate that Pax6-bound regions are mostly enriched with H3K4me2 and H3K4me1 in enhancers and promoters, though H3K4me3 marks only Pax6-containing promoters. The shRNA-mediated knockdown of Pax6 revealed down-regulation of a set of direct target genes, including *Cap2*, *Farp1*, *Pax6*, *Plekha1*, *Prox1*, *Tshz2*, and *Zfp536*. Pax6 knockdown was accompanied by reduced H3K4me1 at enhancers and H3K4me3 at promoters, with little or no changes of the H3K4me2 modifications. These changes were prominent in *Plekha1*, a gene regulated by Pax6 in both lens and retinal pigmented epithelium.

**Conclusions:**

Our study supports a general model of Pax6-mediated recruitment of histone methyltransferases Mll1 and Mll2 to lens chromatin, especially at distal enhancers. Genome-wide data in lens show that Pax6 binding correlates with H3K4me2, consistent with the idea that H3K4me2 PTMs correlate with the binding of transcription factors. Importantly, partial reduction of Pax6 induces prominent changes in local H3K4me1 and H3K4me3 modification. Together, these data open the field to mechanistic studies of Pax6, Mll1, Mll2, and H3K4me1/2/3 dynamics at distal enhancers and promoters of developmentally controlled genes.

**Electronic supplementary material:**

The online version of this article (doi:10.1186/s13072-016-0087-z) contains supplementary material, which is available to authorized users.

## Background

Cellular differentiation is regulated by a combinatorial action of sequence-specific DNA-binding transcription factors and extracellular signaling that results in activation and repression of specific batteries of genes [1–3]. These transcription factors detect regulatory sequences in promoters and enhancers, proximal and distal regulatory regions, respectively. These regulatory elements communicate together through DNA looping [4–6]. Transcriptionally active genes are marked by “open” chromatin domains accessible to nuclease digestions, specific combinations of core histone posttranslational modifications (PTMs), and incorporation of H2A.Z, H3.3 core histone variants into promoter regions [7–9]. In contrast, transcriptionally inactive genes are organized within compact chromatin domains, formation of which is promoted by different sets of core histone modifications. Recent studies have provided novel insights into the structural and functional organization of these processes, including promoter–enhancer looping [3, 10], transcription of enhancer-specific eRNA, and the use of ncRNAs in organizing transcriptional proteins [7, 11]. Nevertheless, the question of how DNA-binding transcription factors influence posttranslational modifications of histones and regulate transcription remains unanswered.

Genome-wide studies of chromatin by ChIP-seq have revealed that there is a relatively small number of core histone PTMs, including H3K4me1, H3K4me3, H3K27ac, and H3K27me3, which can be used as landmarks for navigation through the chromatin landscape. Combinations of these PTMs in genomic regions have been shown to be highly associated with the locations of individual promoters and enhancers [12, 13]. Active promoter regions are occupied by DNA-binding transcription factors and are highly enriched for H3K4me3 and H3K27ac, while active enhancers are marked by a combination of H3K4me1 and H3K27ac. Another PTM, H3K4me2, decorates the majority of active promoters and strong enhancers [13]. Furthermore, clusters of histone PTMs are associated with abundant histone-modifying enzymes, including histone acetyltransferases (HATs) and methyltransferases (HMTs) [14, 15]. How these HATs and HMTs get to developmentally appropriate promoters and enhancers is an open question. Of particular interest is the methylation status of H3K4 residues in histone H3N-terminal tails.

In mammalian cells, H3K4 methylations are catalyzed by a family of six distinct complexes. The Mll/Set1 complexes contain enzymes with an evolutionarily conserved C-terminal catalytical SET domain and an evolutionarily conserved WRAD subcomplex (Wdr5, Rbbp5, Ash2l, and Dpy30). A few additional regulatory proteins discriminate between Mll and Set1 complexes [16]. For example, Set1a/b- and Mll1/2/3/4-containing complexes are different as the Set1 complexes contain additional Cfp1 and Wdr82 subunits [17]. How mono- and dimethylation is “written” onto the fourth lysine of H3 tail differs from how trimethylation as the same residue is generated. H3K4 trimethylation results from promoter-specific H3K4me3 “indexing” during transcription. Specifically, the Wdr82 subunit of Set1a/b complexes binds to the phosphorylated C-terminal domain of RNA polymerase II at the initiation phase of transcription [18]. Alternatively, the CpG-binding protein Cfp1 can recruit Set1a/b complex to the unmethylated CpG promoter regions [19]. Much less is known about the generation of H3K4 mono- and dimethylation. It is possible that the SET domain of these enzymes generates H3K4me1 and that the WRAD subcomplex possesses a “second” HMT activity, raising the possibility that the SET domain containing enzyme generates H3K4me1 and these substrates are dimethylated by the WRAD subcomplex, though the catalytical center of these activities remains unidentified [20].

Pax genes encode DNA-binding transcription factors that function as critical developmental regulators [21]. The Pax6 protein is composed of a bipartite DNA-binding paired domain and an internal homeodomain. Together these domains bind to DNA and might serve as a surface for protein–protein interactions [22]. Pax6 is a key regulator of eye morphogenesis [23, 24] and lens development [25–28]. Pax6 is also highly expressed in the dorsal part of the forebrain and has important functions in neurogenesis and cortical patterning [29]. Pax6^Sey/Sey^ mice are anophthalmic (i.e., lack the eyes) and display a range of abnormalities in other organs, including the brain, olfactory system, and pancreas [30]. The homozygous deletion of Pax6 in the prospective lens ectoderm blocks lens induction [31]. The heterozygous *Pax6*^+*/*−^ lens placodes are composed of reduced cell numbers [32] and subsequently develop into lenses of reduced size with subtle structural abnormalities [27, 28, 30]. Interestingly, simultaneous deletion of CBP and p300 HATs in the prospective lens ectoderm phenocopies defects found in Pax6 null ectoderm [33]. This phenocopying provides a mechanistic link between early roles of Pax6, acetylation of H3K18 and H3K27, and chromatin remodeling during embryogenesis [33]. Genetic studies of Pax6 have revealed a multitude of functions during mouse embryonic development [27, 34], including roles as a dual transcriptional activator and repressor [35, 36]. Pax6-mediated gene regulation is dosage sensitive; *Pax6*^Sey/+^ mice are viable, they have smaller and developmentally defective eyes [37], and their transcriptome is moderately disrupted [33, 38]. Gene reporter assays have also shown that Pax6 has concentration-dependent modes of transcriptional activation and repression [39]. Unlike genetic studies of Pax6 in lens [31, 32, 40–42] and its DNA-binding activities [35, 43, 44], the understanding of Pax6-interacting proteins is in its infancy [22].

In the present study, we aimed to extend the understanding of the molecular mechanisms of Pax6-mediated gene activation and repression [45] by identifying chromatin remodeling activities associated with Pax6. Using an in vitro assay, we detected a histone H3K4 HMT activity enriched in Pax6-specific immunoprecipitates. Subsequent proteomic studies identified Mll1, Mll2, and Set1a in these materials. ChIP-seq data revealed that Pax6 co-localized with H3K4me1/2 in distal enhancers and H3K4me1/2/3 in proximal promoters. Reduction of Pax6 expression in cultured lens cells identified hundreds of differentially expressed genes, including seven positively regulated Pax6-direct targets (*Cap2, Farp1, Pax6, Plekha1, Prox1, Tshz2*, and *Zfp536*). Partial reduction of Pax6 expression resulted in reduced abundance of H3K4me1 in distal enhancers and of H3K4me3 in promoter regions at the genome-wide level.

## Results

### Pax6 is associated with H3K4 methylation activity

**Fig. 1:**
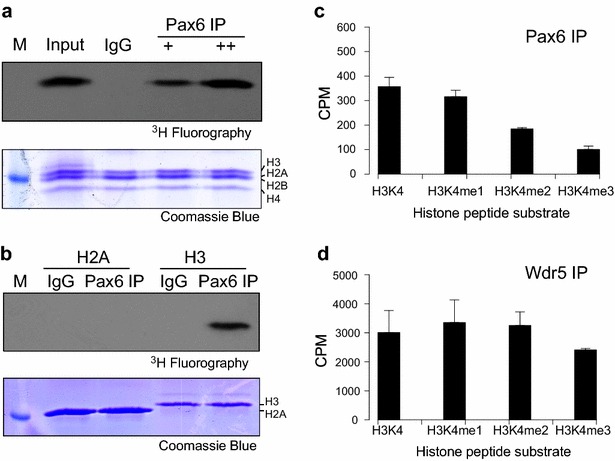
Pax6-immunoprecipitates contain histone methyltransferase activities specific for recombinant histone H3. **a** In vitro HMT assay using recombinant histone octamers. The Pax6-immunoprecipitates were used at 1x (+) and 2x (++) amounts. Input represents the lens cell nuclear extract. IgG-immunoprecipitates were used as a control. **b** In vitro HMT assay using recombinant H2A and H3 histones. IgG-immunoprecipitates were used as a control. **c** In vitro HMT assay using unmodified, mono-, di- and trimethylated H3K4 peptides (residues 1–20) in the presence of Pax6-immunoprecipitates. **d** In vitro HMT assay using unmodified, mono-, di- and trimethylated H3K4 peptides in the presence of Wdr5-immunoprecipitates. The HMT activities of control IgG-immunoprecipitates were subtracted in both **c**, **d**. (*error bars* = ±s.d.)

To test our hypothesis that transcriptional regulation by Pax6 involves the regulation of histone methylation, we first immunoprecipitated Pax6 proteins from nuclear extracts of mouse lens epithelial cells (αTN4). We used Pax6-specific antibodies and tested the enriched proteins by in vitro HMT assay performed in the presence of labeled [^3^H] S-adenosyl methionine as methyl group donor and recombinant histone octamers as the substrates. We found that Pax6-, but not control IgG-immunoprecipitates, were associated with HMT activity (Fig. 1a). To distinguish between the histones H3 and H2B that closely migrate on the SDS-PAGE, we performed additional HMT assays using the individual recombinant H3 and H2B histones. We found that methylation was specific for histone H3 (Fig. 1b). To identify the potential methylation site and distinguish the methylation status of H3, we conducted an in vitro HMT radiometric filter assay using H3N-terminal peptides (residues 1–20) with an unmodified, mono-, di-, or trimethylated lysine 4 (i.e., H3K4, H3K4me1, H3K4me2, and H3K4me3 histone tail mimics). Pax6-containing immunoprecipitates catalyzed methylations of these four peptides as various levels. We found comparable methylation efficiencies between unmethylated and monomethylated peptides (Fig. 1c). In contrast, the HMT activity was reduced when dimethylated histone tail mimics were used, and the lowest incorporation of the methyl donor group was detected with the trimethylated peptides. We next evaluated Wdr5-containing immunoprecipitates and found that the HMT activities were much higher (Fig. 1d), most likely as Wdr5 is a common subunit of multiple Mll/Set1 complexes. These data suggest that this reconstituted in vitro methylation Pax6-containing system possesses the ability to modify monomethylated substrates and that the system can utilize H3K4me1 peptide mimics for additional methylations and raise the possibility that Mll/Set1 complexes may be present in Pax6-containing immunoprecipitates.

### Pax6-immunoprecipitates from lens nuclear extracts contain Set1a, Mll1, and Mll2

**Fig. 2:**
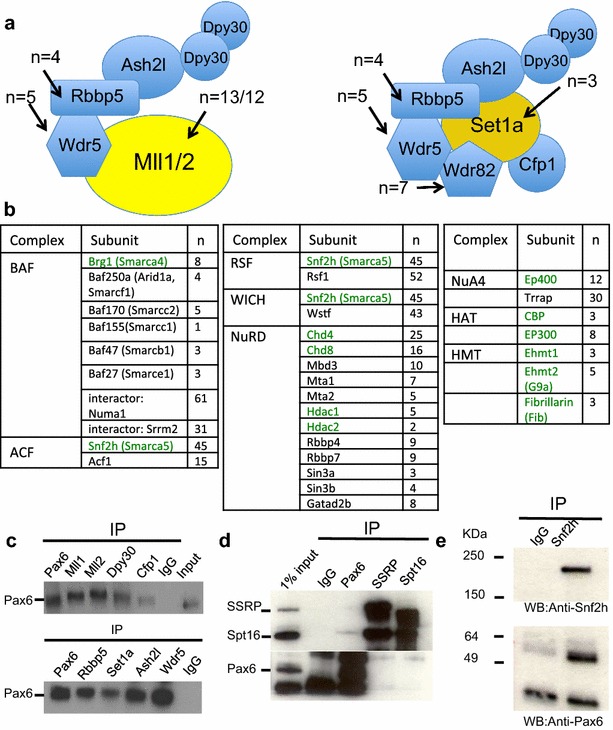
Identification of individual protein components in Pax6-containing immunoprecipitates. **a** Subunit structure of Mll1/2 and Set1a complexes and number of specific peptides (*n*) of these subunits identified by LC–MS/MS. **b** Pax6-immunoprecipitates contain additional subunits of multiple chromatin-modifying and remodeling complexes, including BAF, ACF, RCF, WICH, NuRD, NuA4, HAT, and HMT. The catalytical subunits of these complexes are shown in *green*. **c** Co-IP validation of Pax6 in immunoprecipitates obtained using Mll1, Mll2, Set1a, Wdr5, Rbbp5, Cfp1, Ash2l, and Dpy30 antibodies. **d** Co-IP validation of the FACT complex subunits Ssrp and Spt16 in Pax6-immunoprecipitates. **e** Co-IP validation of the Snf2h (Smarca5) in Pax6-immunoprecipitates. IgG-immunoprecipitates were used as control. Protein markers are shown in kDa

Mammalian genomes encode at least six different protein complexes that are known to methylate H3K4 residues. To identify the H3K4 methylase and other proteins associated with Pax6, we employed a non-biased proteomic approach. By immunoprecipitating with Pax6 antibodies, we purified “Pax6 complexes” and used liquid chromatography-tandem mass spectrometry (LC–MS/MS) to identify Pax6-associated proteins in the nuclear extract prepared from αTN4 cultured lens epithelial cells. In total, we identified 301 protein clusters with a high confidence score as described in “Methods” (Additional file 1: Table S1). The majority of the identified proteins belong to the functional groups of chromatin modifiers, chromatin remodelers, RNA processing, or DNA-binding proteins (Additional file 1: Table S1). Importantly, the chromatin modifiers identified include Mll1, Mll2, and Set1a enzymes and their associated proteins (Fig. 2a). Other notable chromatin modifiers and remodelers include ISWI, SWI/SNF, NuRD complexes, p300, and CBP HATs (Fig. 2b). It was previously shown that Pax6 interacts with p300 in cell extracts of cultured pancreatic α-cells [46], ATP-dependent catalytical subunit of SWI/SNF complexes Brg1 (Smarca4) in extracts from mouse adult neural stem cells, and BAF170 (Smarcc2) in mouse cerebral cortex [47, 48]. In addition, the Brg1/Pax6 complexes were detected in co-transfected 293T cells [49]. Immunoprecipitations using Mll1, Mll2, and Set1a antibodies revealed the presence of Pax6 proteins (Fig. 2c). We further identified all common Mll complex subunits, i.e., Wdr5, Rbbp5, Ash2l, and Dpy30, by independent co-IPs followed by western blots (Fig. 2c). In addition, we validated the presence of both subunits of the histone chaperone complex FACT, Ssrp and Spt16 [50], which remodels nucleosomal structure to facilitate RNA polymerase II movement through nucleosomes (Fig. 2d). Finally, we found that fragments of Snf2h (Smarca5), and its three regulatory subunits Rsf1, Wstf, and Acf1 (Fig. 2b), forming the binary RSF1, WICH, and ACF chromatin remodeling complexes, respectively [51], were highly abundant in Pax6-immunoprecipitates. The presence of Snf2h in Pax6-immunoprecipitates was also validated by co-IP westerns (Fig. 2e). Consistent with the role of Pax6 in transcriptional repression [35, 36], all components of the histone deacetylase-containing NuRD complexes [52] were also found (Fig. 2b). It is worth noting that two abundant lens nuclear proteins, menin-binding protein Psip1 (alternate names: LEDGF, p75) [53] and Ncoa6 (alternate names: AIB3, ASC2, RAP250, Trbp) [54], were not found (Additional file 1: Table S1). Both Psip1 and Ncoa6 are substoichiometric subunits of Mll1/2 and Mll3/4 complexes [17], respectively. Taken together, these proteomic studies coupled with in vitro methyltransferase assays support the idea that Pax6-Mll1, Pax6-Mll2, and Pax6-Set1a complexes exist in lens cell nuclear extracts.

### Distribution of histone PTMs at promoters and enhancers in lens chromatin

**Fig. 3:**
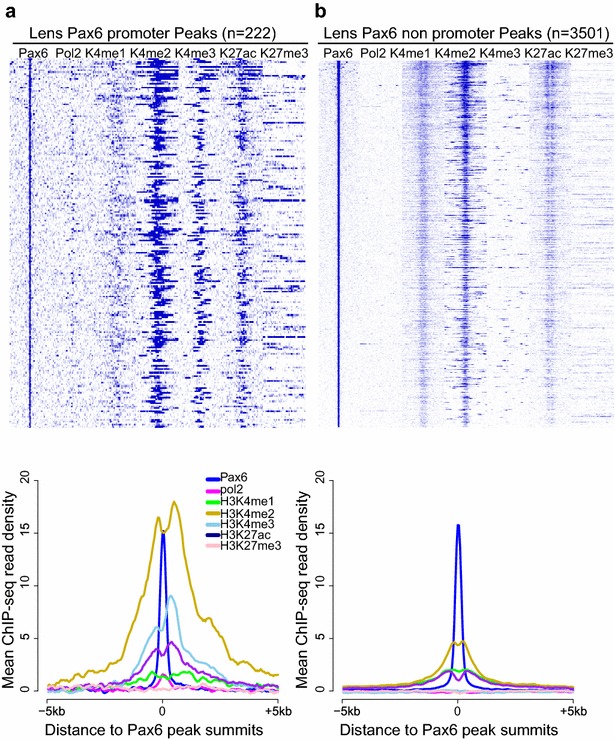
Lens Pax6 promoter and non promoter peaks show different histone modification patterns in mouse lens chromatin. **a** Pax6 promoter peaks are co-localized with H3K4me2, H3K4me3 and H3K27ac in mouse lens chromatin. **b** Lens Pax6 non-promoter peaks are co-localized with H3K4me1, H3K4me2, and H3K27ac. The heatmap shows read densities in 50-bp bins from ±5 kb of the Pax6 peak summits. Pax6, RNA polymerase II, H3K4me1, H3K4me2, H3K4me3, H3K27ac, H3K27me3 ChIP-seq data in lens tissue are shown. The *lower panels* show mean ChIP-seq read densities from −5 to +5 kb around Pax6 peak summits. The *rows* in the heatmaps were sorted by the Pax6 signals (likewise in Figs. 5, 7).

The biochemical association between Pax6 and enzymes that catalyze the methylation of H3K4 residues prompted us to examine the distribution of H3K4me1, H3K4me2, and H3K4me3 in regions of lens chromatin occupied by Pax6. Previously, we had mapped H3K4me1, H3K4me3, H3K27ac, H3K4me3, and RNA polymerase II in newborn lens chromatin [45]. Here we also analyzed the localization of H3K4me2 at 222 Pax6-bound promoters and proximal to 3501 non-promoter Pax6-containing peaks (Fig. 3). In the promoters (Fig. 3a), the normalized signal intensities for H3K4me2 around Pax6-bound sites were higher compared to H3K4me3 and H3K27ac levels. The “peaks” in the H3K4me2 and H3K4me3 profiles were shifted from the Pax6 summits, while reduced nucleosomal density was indicated by small valleys near the Pax6 peaks (Fig. 3a). In contrast, in the non-promoter regions the profiles for H3K4me1, H3K4me2, and H3K27ac were symmetrical around the Pax6-binding sites, but also showed a reduction at the center of Pax6 peaks (Fig. 3b). By computing the correlation of Pax6 and H3K4me1/2/3 ChIP-seq read densities across Pax6-binding sites (±5 kb of Pax6 peak summits), we found that Pax6 occupancy was significantly correlated with H3K4me in both promoters and distal regions. The Pearson’s correlation coefficients (*r*) for the promoter Pax6 peaks were 0.30 (*p* = 3.8e-6) for H3K4me1, 0.31 (*p* = 1.96e-6) for H3K4me2, and 0.24 (*p* = 2.5e-4) for H3K4me3, while the coefficients were 0.38 (*p* = 4.73e-15), 0.26 (*p* = 2.64e-55), and 0.11 (*p* = 1.17e-10) for the non-promoter Pax6 peaks, respectively. Together with the data shown in Fig. 3, these quantification analyses indicate that Pax6 occupancy shows the largest correlation with H3K4me1 enrichment but also agree with previous genome-wide studies implicating H3K4me2 as a marker of tissue-specific gene regulation [55] and transcription factor binding regions [56].

### Identification of Pax6 sites in cultured lens epithelial cells

**Fig. 4:**
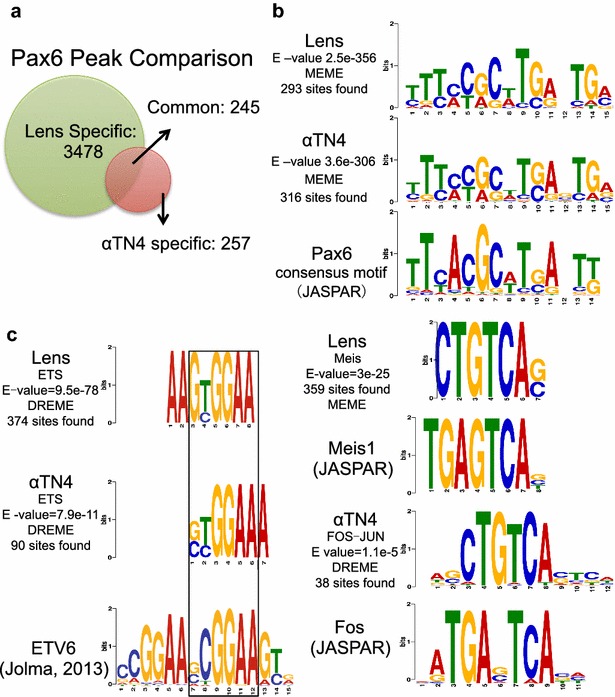
Pax6-binding site analysis and identification of enriched motifs around Pax6 peak summits. **a** 502 Pax6 peaks were identified in αTN4 chromatin, including 245 shared with the newborn mouse lens chromatin. **b** 502 Pax6 peaks identified in αTN4 chromatin are enriched with Pax6 motifs similar to those identified from 3723 Pax6 peaks in lens chromatin and by in vitro DNA-binding studies. **c** Additional motifs assigned to Ets, Meis, and AP-1 families of transcription factors were also identified at the Pax6 peaks. The regions examined are defined as ±100 bp under the Pax6 peak summits

To gain mechanistic insight into Pax6 binding and H3K4 methylations, we established a cell culture system suited to the down-regulation of Pax6. We analyzed Pax6 binding in αTN4 lens cells used in biochemical studies described above by ChIP-seq and found 502 peak regions. We identified 245 of them as being common to primary lens and cultured lens cells (Fig. 4a). To demonstrate the specificity of these peaks, we found significant enrichment of Pax6 consensus motifs within these Pax6 peaks (Fig. 4b) [35, 43, 45]. It is worth noting that we found additional common *cis*-motifs enriched at Pax6-bound promoters and enhancers, including Ets, Meis, and AP-1 (Fos-Jun)-binding sites (Fig. 4c). Individual members of these families of transcription factors, including c-Jun, Etv5, Meis1, and Meis2, regulate lens development [25].

**Fig. 5:**
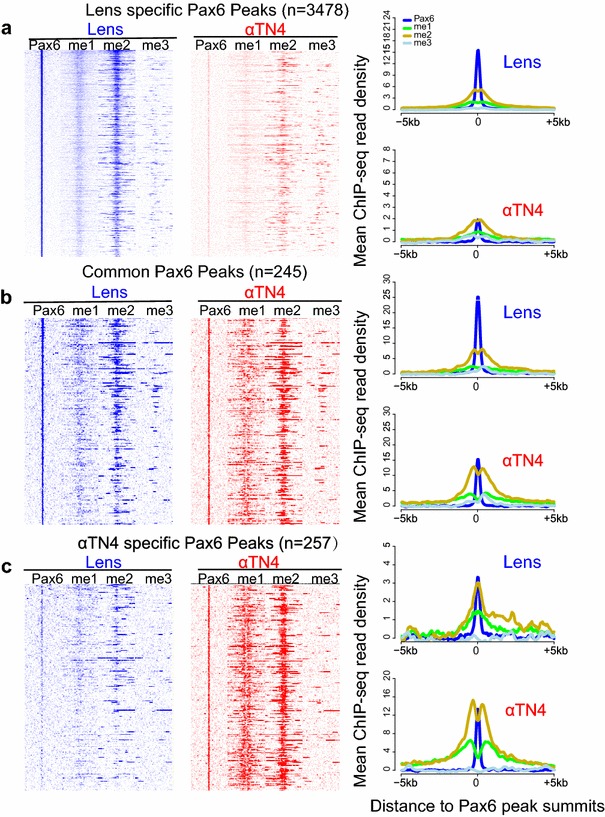
Lens-specific and common Pax6 peaks of mouse lens chromatins show similar histone modification patterns. **a** Lens-specific Pax6 peaks are co-localized with H3K4me1, and H3K4me2 in mouse lens tissue. Heatmap shows read density in 50-bp bins from −5 to +5 kb of the peak summits at lens-specific Pax6 peaks (*n* = 3478). **b** Common Pax6 peaks are co-localized with H3K4me1, and H3K4me2 in both lens tissue and αTN4 cells. Heatmap shows read density in 50-bp bins from −5 to +5 kb of the peak summits at Pax6 common peaks between lens tissue and αTN4 cells (*n* = 245). **c** αTN4 specific Pax6 peaks are co-localized with H3K4me1, and H3K4me2 in αTN4 cells. The *right panel* shows mean ChIP-seq read density for all ChIP-seq data from −5 to +5 kb around Pax6 peak summits

We next determined the distribution of H3K4me1, H3K4me2, and H3K4me3 in αTN4 lens chromatin. Based on Pax6 ChIP-seq data (Fig. 4a), we separated Pax6 peaks into lens-specific (*n* = 3478), αTN4/lens “common” (*n* = 245) peaks, and αTN4-specific peaks (*n* = 257) (Fig. 5). Interestingly, we found that lens-specific and αTN4-specific Pax6 peaks showed a greater enrichment of H3K4me1/2 in the specific cell types where Pax6 binding was detected (Fig. 5a,c), whereas “common” Pax6 peaks displayed similar H3K4me1/2 enrichment in both cell types (Fig. 5b). These results further support the conclusion from Fig. 3 that Pax6 binding is highly correlated with H3K4me1 and H3K4me2, i.e., enhancer regions. These studies also indicate that Pax6-direct target genes in αTN4 cells may function as models to probe the relationship between Pax6 binding and H3K4 methylations.

### Pax6 knockdown and gene expression changes

**Fig. 6:**
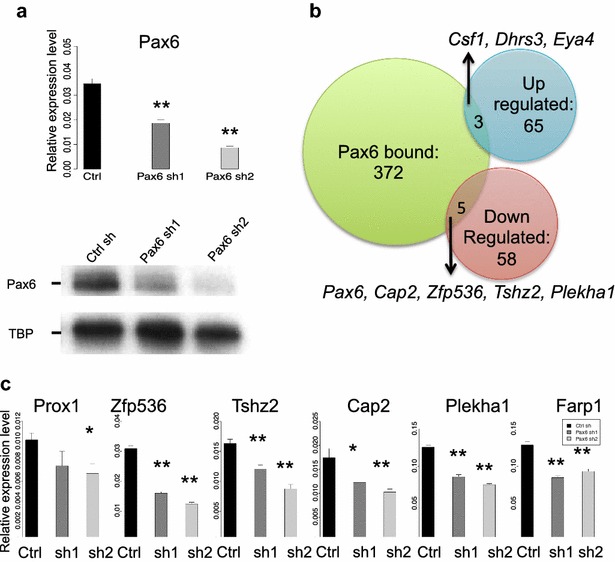
Analysis of gene expression in Pax6 shRNA lens cell lines. **a** Knockdown of Pax6 by lentivirus shRNA (sh1 and sh2). *Upper panel* qRT-PCR. *Lower panel* western immunoblot. **b** Overlap of Pax6-bound genes and differentially expressed genes. Differentially expressed genes were detected by RNA-seq. **c** qRT-PCR validation of Pax6 positively regulated genes: Cap2, Farp1, Pax6 (see **a**), Plekha1, Prox1, Tshz2, and Zfp536. *p* values <0.05 by Student’s *t* tests are labeled as *, while *p* values <0.01 were labeled as **

To test the link between Pax6 and methylation of H3K4, we used shRNA-mediated Pax6 knockdown (KD) in αTN4 cells to identify genes regulated by Pax6. To achieve this goal, we established two independent stable Pax6 KD αTN4 cell lines with two different shRNA constructs. The knockdown efficiency was examined by qRT-PCR and immunoblotting. There was an 80 % reduction of Pax6 mRNA and protein levels in the Pax6 sh2 line, but only a 60 % reduction in the Pax6 sh1 line (Fig. 6a). Neither of these engineered cell lines displayed any obvious defects in morphology or growth rate.

To find which genes were affected by reduced Pax6 levels, we performed RNA analysis in both control and Pax6 sh1 cells to identify differentially expressed genes that were sensitive to Pax6 reduction. We pooled two biological replicates each and performed a comparative analysis by RNA-seq. In total, we found 131 genes significantly differentially expressed in sh1 Pax6 KD cells, including 68 up- and 63 down-regulated transcripts. Among these genes, a group of seven genes, including *Cap2*, *Farp1*, *Pax6*, *Plekha1*, *Prox1*, *Tshz2*, and *Zfp536*, were both bound by Pax6 and differentially expressed in both Pax6 sh1 and sh2 cells (Fig. 6b). We next evaluated expression of these genes using qRT-PCR (Fig. 6c). Expression of Cap2, Pax6, Plekha1, Tshz2, and Zfp536 transcripts was also significantly reduced in Pax6 KD cells (*p* = 0.001 by Fisher’s exact test, Fig. 6c). In contrast, three genes, including *Csf1*, *Dhrs3*, and *Eya4*, were up-regulated as a result of Pax6 depletion. It is worth noting that Prox1 and Pax6 have already been shown to be direct Pax6 targets in newborn lens [45].

### Reduction of Pax6 expression changes levels of H3K4me3 in promoters and H3K4me1 in distal regions

**Fig. 7:**
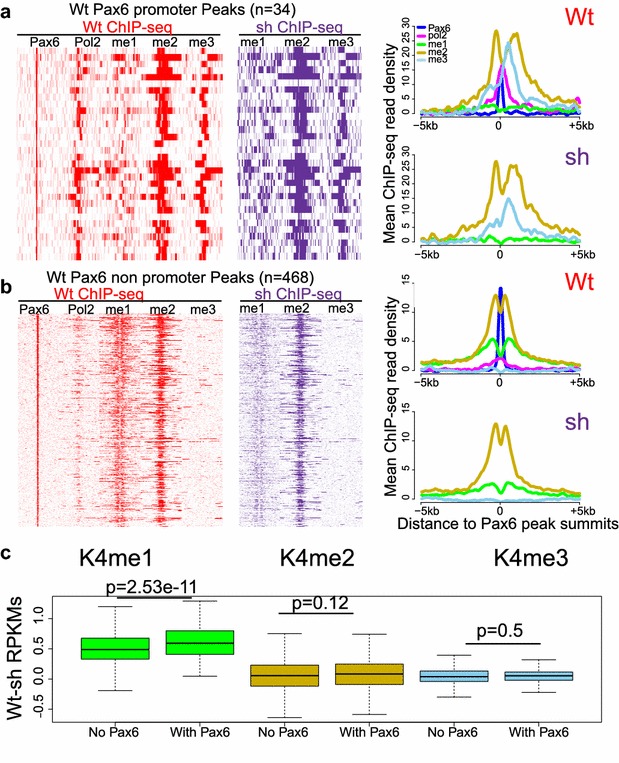
Disruption of histone methylation patterns around Pax6 peaks in Pax6 KD cell line sh2. **a** αTN4 Pax6 promoter peaks (*n* = 34) showed decreased H3K4me1 and H3K4me3 modification but no change of H3K4me2 in Pax6 KD cell line (vs wt). Pol2 data are also shown to indicate promoters. **b** Non-promoter αTN4 Pax6 peaks showed decreased H3K4me1 modification in Pax6 KD cell line (*n* = 468). Heatmaps show read densities from ±5 kb of the Pax6 peak summits, sorted by the Pax6 ChIP-seq signal in WT, with the profiles of mean ChIP-seq read densities plotted in the right (*top* for WT and *bottom* for shPax6 data). **c** Changes (wt vs Pax6 KD) of H3K4me1/2/3 at two groups of enhancers (used WT H3K4me1 peaks as a proxy here) separated by their overlap with Pax6 peaks. Upon Pax6 KD, the enhancers with Pax6 binding showed a greater reduction of H3K4me1 (but no change in H3K4me2/3) than those without Pax6 binding in WT. The *boxplots* show RPKM (reads per kb peak per million ChIP-seq reads) differences between control and Pax6 KD αTN4 cells; the RPKMs were computed for at ±5 kb of the centers of H3K4me1 peaks

To test how reduced expression of Pax6 influences H3K4 methylation, we conducted ChIP-seq studies of H3K4me1, H3K4me2, and H3K4me3 in control and Pax6 KD sh2 αTN4 cells (Fig. 7). No Pax6 peaks were called from the Pax6 KD cells by our analysis (data not shown), so we analyzed all Pax6 peaks in the control αTN4 cells. In the promoters (*n* = 34, with corresponding pol2 enrichment), we found a strong reduction in H3K4me3 and a weak reduction in H3K4me1 signals but no changes in H3K4me2 (Fig. 7a). In the distal non-promoter regions (*n* = 468), the major difference was reduced H3K4me1 abundance (Fig. 7b). To evaluate the statistical significance of H3K4me1 reduction in non-promoter Pax6 peaks that may function as enhancers, we decided to analyze the changes of H3K4me1/2/3 read densities between WT and Pax6 KD αTN4 cells at all “enhancers,” which were identified as H3K4me1 peaks in the WT chromatin. As evaluating by a Mann–Whitney U statistical test, we found that the reduction of H3K4me1 in the Pax6-bound enhancers was statistically significantly higher than that in the enhancers without Pax6 binding (*p* = 2.53e-11), while the changes of H3K4me2/3 were not significant (Fig. 7c), indicating that H3K4me1 reduction at distal enhancers is related to reduced Pax6 occupancy in Pax6 KD αTN4 cells. Similarly, we also compared promoters with or without Pax6 binding; however, we found the marked reduction of H3K4me3 in the promoters (Fig. 7a) was not statistically significant (*p* = 0.30), which may be due to the small numbers of promoters bound by Pax6 (*n* = 34).

### Plekha1 is regulated by Pax6 in mouse αTN4 and RPE cells

To illustrate the connections between Pax6 and H3K4me1 and H3K4me3, we focused on pleckstrin homology domain containing, family A (phosphoinositide binding specific) member 1, *Plekha1*. The human *PLEKHA1*-*ARMS2*-*HTRA1* gene cluster is on chromosome 10; GWAS studies have implicated this cluster in the pathogenesis of age-related macular degeneration (AMD) [57], a disease caused by dysfunctional retinal pigmented epithelium (RPE). Differentiation of RPE cells is regulated by the transcription factors Pax6, Otx2, and Mitf [58]. Recent studies have shown that Otx2 [59, 60] regulates Plekha1 expression during mouse ES cell differentiation [61] and that binding of Otx2 was found in the Plekha1 gene in the adult mouse neuroretina [62].

**Fig. 8:**
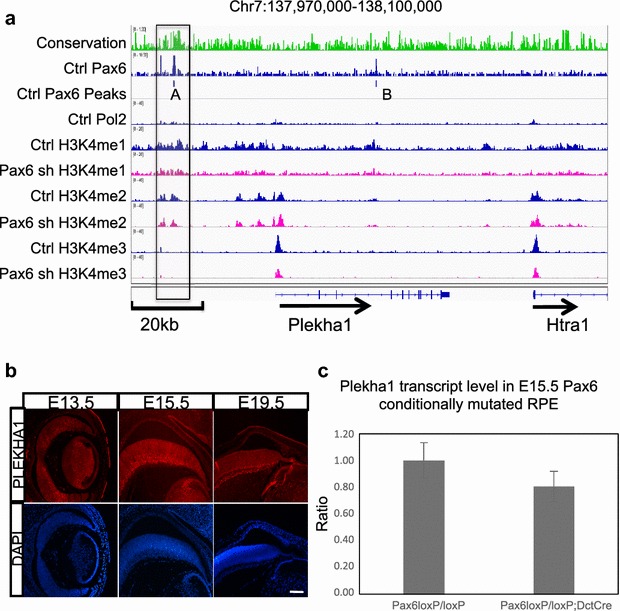
Regulation of Plekha1 gene expression by Pax6. **a** Reduced Pax6 binding affects H3K4me1 and H3K4me3 patterns at Plekha1 enhancer and promoter regions. Pax6, H3K4me1, H3K4me2, H3K4me3, and Pol II ChIP-seq signal at *Plekha1* locus in cultured lens cell chromatin. The mouse *Plekha1* locus is shown including the portion of the downstream *Htra1* gene. The evolutionary conservation (*upper track*-*green color*) and predicted Pax6-binding sites A (see Fig. S2 for details) and B are indicated. **b** Expression of Plekha1 proteins in mouse embryonic eye. Note that in the lens transitional zone and other cell types, Plekha1 (*red*) is found in the nuclei (DAPI stainings, *blue*). *Scale bar* is 100 µm. **c** qRT-PCR expression of Plekha1 in the mouse E15.5 RPE of control *Pax6* ^*loxP/loxP*^ and mutant *Pax6* ^*loxP/loxP*^ *; DctCre* demonstrated a fold change of 0.81 (*pV* = 0.005, *n* = 3). Spearman’s correlation between Pax6 and Plekha1 is 0.76 (*pV* = 0.037, *n* = 3)

In the mouse *Plekha1* locus two Pax6-containing peaks were identified in the evolutionarily conserved 5′-distal region ~27-kb upstream (region A) and in the third intron (region B) in control αTN4 cell chromatin (Fig. 8a). Two predicted Pax6-binding sites as well as accompanying Maf- and Sox-binding sites in region A are shown in Additional file 2: Fig. S2. Importantly, region A is marked by abundant H3K4me1 and H3K4me2 as well as RNA polymerase II suggesting a putative enhancer. In Pax6 KD αTN4 cells, both H3K4me1 and H3K4me2 signals around region A are reduced and accompanied by reduced abundance of H3K4me3 in the Plekha1 promoter (Fig. 8a).

To gain additional insights into Plekha1 gene expression, we determined its expression in the mouse embryonic eye (stages E13.5, E15.5, and E19.5). We found Plekha1 proteins showed nuclear expression throughout the eye, most notably in the corneal epithelium, lens, and neuroretina (Fig. 8b). Finally, in order to examine the significance of Pax6’s regulation of Plekha1 outside of the lens, we tested this system in the RPE. E15.5 RPE from mice with tissue-specific Pax6 depletion [58] were analyzed by qRT-PCR and demonstrated reduction of Plekha1 transcript level (Fig. 8c). Taken together, these data identify Plekha1 as a novel dosage-sensitive direct target of Pax6 in lens and RPE cells.

## Discussion

Sequence-specific DNA-binding transcription factors regulate gene expression by controlling the activity of enhancer regions. What molecular mechanisms are used for this regulation is a major unanswered question in the field of gene regulation and embryonic development. It has been proposed that the recruitment of chromatin remodeling enzymes/complexes by DNA-binding factors elicits local changes in chromatin structure that either promote or inhibit gene expression. A combination of genetic and functional studies has shown that a sparse number of transcription factors, including FoxA1, Gata1, HNF4α, MyoD, Mitf, Nrl, PU.1, Pax5/BSAP, Pax6, Runx2, and Sox9, function as molecular switches to control cell-fate decision steps. Within this group, Pax6 functions during the earliest stages of eye development in both ectoderm- and neuroectoderm-derived progenitor cells and regulates many subsequent steps of eye morphogenesis. As Pax6 functions as a dual transcriptional activator and repressor [25], we reasoned that an unbiased identification of its associated proteins and enzymatic activities toward core histone proteins would provide novel insights into mechanisms of Pax6-mediated gene control during embryonic development. The positively acting chromatin remodeling complexes/enzymes identified here include Mll1, Mll2, and Set1a HMT complexes and CBP and EP300 HATs. The identification of the NuRD complex in Pax6-immunoprecipitates may explain how Pax6 functions as a transcriptional repressor. The dual role of Pax6 in activation and repression could be also mediated through recruitment of SWI/SNF and ISWI chromatin remodeling complexes [47–49]. We have shown earlier that CBP and p300, Brg1 (Smarca4), and Snf2 h (Smarca5) regulate lens induction [33] and differentiation [63, 64].

The interactions between Pax6 and Mll/Set1 complexes are further supported by our findings that Pax6-containing immunoprecipitates contain important regulatory subunits such as the WRAD subcomplex and catalyze in vitro methylation of H3 core histones and H3-derived histone tail mimetics. Although the methylation reactions are markedly reduced when H3K4me3 substrates are used with Pax6-immunoprecipitates, the “residual” activity detected raises the possibility that the other lysine or arginine residues present are also methylated in this system, though direct proof remains to be obtained.

To gain insights into Pax6-dependent histone PTMs, we studied the landscapes of H3K4 methylation in three systems: newborn lens and control and Pax6 KD αTN4 lens cells. The analysis of lens chromatin identified a preferred association between Pax6 binding and H3K4me2. Nevertheless, upon Pax6 KD expression, the abundance of distal H3K4me1 and promoter H3K4me3 modification, but not H3K4me2 modification, was reduced at a genome-wide scale. These data imply that Pax6 may recruit Mll1 and Mll2 to the distal regions and Set1a to the promoters. These protein recruitments could catalyze H3K4me1 and H3K4me2 modifications and generate H3K4me3 residues. Although the Set1a/b complexes bind to the phosphorylated C-terminal domain of RNA polymerase II via the Wdr82 subunit at the initiation phase of transcription, our data suggest that Pax6 may also recruit a fraction of the Set1a complex to the promoter prior the onset of transcription. Additional studies will be needed to determine the localizations of Mll1, Mll2, and Set1a enzymes in lens chromatin (by ChIP-seq when antibodies are available and/or by engineering the αTN4 to insert in frame epitope tags into genes encoding the Mll/Set1a-specific subunits). At the level of individual genes, we document changes at Plekha1’s distal enhancer. In addition, we show that Pax6 regulates Plekha1 in RPE cells and we establish for the first time its expression domains in the mouse embryonic eyes.

Although ten genes with disrupted regulation were found in the present Pax6 KD αTN4 system, it is important to stress that the reduction of Pax6 expression was in the range of inactivating one functional Pax6 copy (haploinsufficiency) and this reduction in vivo generates only subtle defects in the lens. We restrained ourselves from reducing the Pax6 protein to ~10–15 % of normal levels, as we were concerned that the engineered αTN4 cells would lose their cell-type identity.

To better understand our Pax6 KD system, we considered a few “indirect” possibilities: that depletion of Pax6 could affect expression of subunits comprising the Mll/Set1 complexes, or that Pax6 could be “globally” involved in controlling the levels of H3K4 methylations. To test the first possibility, we examined the protein levels of Ash2l and Rbbp5 in both control sh and Pax6 sh2 cells by immunoblotting. We normalized the protein levels to TATA-box-binding protein (TBP) and did not find any global changes (Additional file 2: Fig. S1a). To examine whether there were any cellular H3K4me changes in Pax6 KD cells, we compared the H3K4 methylation levels from the whole cell lysates of control and Pax6 KD sh2 cells by immunoblotting. Individual H3K4me1, H3K4me2, and H3K4me3 signals were normalized to TBP, and no notable changes were found (Additional file 2: Fig. S1b), indicating that a reduction of Pax6 expression did not affect the net cellular activity of H3K4 methylases. We concluded that reduction of Pax6 expression does not affect expression of two common WRAD subunits and global levels of methylated histones.

**Fig. 9:**
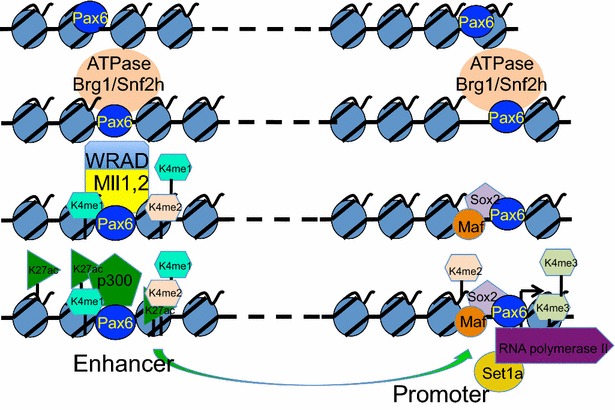
A general model of enhancer-dependent transcriptional activation by Pax6 through recruitment of chromatin-modifying and remodeling complexes. The present data coupled with earlier studies on Pax6 suggest a general model that explains chromatin features near Pax6-bound peaks. The Pax6/Brg1- and Pax6/Snf2h-containing ATP chromatin remodeling complexes are initially assembled in the enhancer and promoter regions. Pax6 is then joined by additional DNA-binding factors (not shown). In subsequent stages, enhancer-bound Pax6 recruits Mll1/2 complexes and the region is marked by H3K4me1 and H3K4me2, followed by recruitment of p300, and generation of H3K27ac. Similarly, at the promoter regions, binding of Pax6 facilitates recruitment of cooperating DNA-binding factors (shown: Maf and Sox2), followed by various chromatin-modifying and remodeling activities, and formation of physical contacts between these assemblies mediated by DNA looping. As the transcription commences, Set1a traveling with the RNA polymerase will convert the unmodified and partially methylated H3K4 residues into the high density of H3K4me3

The molecular mechanisms underlying the genesis of tissue-specific enhancers, the significance of individual and combined histone PTMs, and the “writing” order and “reading” recognition of histone PTMs, remain poorly understood, especially in the context of developmentally regulated genes. Enhancers can be viewed as highly organized chromatin domains primarily organized by sequence-specific transcription factors. During and/or following their formation, the distal enhancer domains physically contact the promoter-bound protein–DNA complexes, and promoter–enhancer loops are established to facilitate efficient recruitment of the basal transcriptional machinery [10]. The birth of enhancers is thought to involve recruitment of multiple chromatin remodeling systems by the “pioneering” transcription factors [65, 66]. The “net” effect of these DNA–protein–protein interactions is the generation of enhancer-specific patterns of core histone PTMs, including H3K4me1 and H3K27ac [13, 67] and the establishment of “open” chromatin/nucleosome-free regions [66, 68, 69]. Pax6 functions as a highly selective molecular switch that activates gene expression in different cell types and represses those same genes in other cell types [25]. Our earlier studies have shown that Pax6 binds 2/3 of common sites in two distinct lens and forebrain chromatins [45]. This finding raises the possibility that Pax6 functions as a pioneering factor. Previous studies have shown that Pax6 binds with p300 HAT [46] and SWI/SNF complexes via direct binding to Brg1, Brm, Baf155, and Baf170 [47–49]. The present data add Mll1, Mll2, and Set1a to the list of Pax6-associated chromatin-modifying enzymes and suggest a number of additional novel chromatin remodeling complexes are linked to Pax6, such as ACF, RSF, WICH, and NuRD, which explain the dual roles of Pax6 as transcriptional activator and repressor (Fig. 9). Taken together, Pax6 possesses many activities attributed to pioneering factors; nevertheless, additional studies are needed to find whether Pax6 can access its target sites in chromatin independently on other DNA-binding transcription factors.

At present, few DNA-binding transcription factors are known to bind Mll-containing HMT complexes. The Pax2, Pax3, and Pax7 factors have been shown to bind Mll3/4 complexes via the adaptor protein PTIP [70] or through distinct adaptor protein Pax3/7BP (official name: Paxbp1) [71]. Recently, the developmental regulators MafA [72], Oct4 [73], Pitx2 [7], and Tbx1 [75] have also been shown to interact with specific Mll complexes. Neither PTIP nor Paxbp1 was detected among the 301 Pax6-interacting clusters. We propose that the common property of multiple Pax transcription factors is to direct recruitment of Mll HMT complexes.

The most common partners of Pax6 in tissue-specific gene control in lens include bZIP protein c-Maf, nuclear receptor complex RARβ/RXRβ, and HMG-box Sox2 [39, 76] and are all known to bind p300 and CBP HATs [77]. Thus, the “master” role of Pax6 in embryonic development can be explained in molecular terms by its ability to recruit a full complement of positively acting chromatin remodelers (e.g., HATs, HMTs, and ATP-dependent remodelers) and to provide additional service through recruitment Mll1 and Mll2, which methylate the enhancers. Future studies will be aimed to test these Pax6 protein–protein interactions and their role in enhancer-mediated tissue-specific gene control.

## Conclusions

This study reveals interactions of Pax6 with multiple chromatin-modifying and remodeling complexes and supports a general model of Pax6-mediated recruitment of histone methyltransferases Mll1 and Mll2 at distal enhancers in lens chromatin. Although genome-wide data in lens show that Pax6 binding correlates with H3K4me2, consistent with the idea that H3K4me2 PTMs correlate with the binding of transcription factors, reduction of Pax6 by shRNA expression induces prominent changes in local H3K4me1 and H3K4me3 modifications. These findings open the field to mechanistic studies of Pax6, Mll1, Mll2, and dynamics of H3K4 methylations at distal enhancers and promoters of developmentally controlled genes in lens and other tissues regulated by Pax6, including forebrain, retina, and pancreas.

## Methods

### Antibodies

Ash2l (Bethyl, A300-489A), Cfp1 (Abcam ab198977), Dpy30 (Bethyl A304-296A), H3K4me1 (Abcam, ab8895), H3K4me2 (Abcam, ab7766), H3K4me3 (Abcam, ab8580), H3K9me2 (Abcam, ab1220), H3K79me2 (Abcam, ab3594), IgG (Millipore 12-370), Mll1 (Bethyl A300-086A), Mll2 (Santa Cruz, H300, sc-292359), Pax6 (Millipore, ab2237), Plekha1 (Novus, NBP1-86967), Rbbp5 (Bethyl, A300-109A), RNA polymerase II (Santa Cruz, N-20), Set1a (Bethyl, A300-289A), Snf2h (Active Motif, 39543), Spt16 (Biolegend, 607001), Ssrp (Biolegend 609702), TBP (Abcam, ab51841), vinculin (Abcam, ab129002), and Wdr5 (Bethyl A302-429A) were used.

### Immunoprecipitation analysis

Mouse lens epithelial cell line αTN4 was used for immunoprecipitation assays. All procedures were done at 4 °C. Nuclear extracts were prepared as described [78]. Protein G Dynabeads (Invitrogen) were used for immunoprecipitation. The beads were block with 1 % BSA before the day of immunoprecipitation. On the day of immunoprecipitation, nuclear extracts were dialyzed into the BC200 (20 mM HEPES, pH 7.9, 0.2 mM EDTA, 0.5 mM DTT, 20 % glycerol, 0.2 % NP-40, and 200 mM KCl) buffer. For antibody conjugation, 5 µg of Pax6, Wdr5, or control IgG antibodies was incubated with Dynabeads for at least 6 h. For nuclear extract pre-cleaning, 200 µg nuclear extracts were diluted with BC200 to 200 ng/µl and incubated with Dynabeads for 2 h. Pre-cleaned nuclear extracts were incubated with antibody-conjugated Dynabeads for overnight. The beads were washed twice with BC200 and twice with BC500. Finally, the beads were resuspended in 30 µl BC200 and used for subsequent assays.

### Mass spectrometry

Samples processed for LC–MS/MS were subjected to SDS-PAGE and silver stained as described previously [79]. Bands were excised and analyzed on a LTQ linear ion trap mass spectrometer (ThermoFisher Scientific, Waltham, MA) interfaced with a Rapid Separation LC 3000 system (ThermoFisher Scientific) and a TriVersa NanoMate system (Advion, Ithaca, NY). Mgf files were created from the raw LTQ mass spectrometer LC–MS/MS data using Proteome Discoverer 1.3 (ThermoFisher Scientific). The created mgf files were used to search the NCBI database using the in-house Mascot Protein Search engine version 2.4.1 (Matrix Science) with the following parameters: trypsin 2 missed cleavages; fixed modification of carbamidomethylation (Cys); variable modifications of deamidation (Asn and Gln), pyro-glu (Glu and Gln), and oxidation (Met); monoisotopic masses; peptide mass tolerance of 2 Da; product ion mass tolerance of 0.6 Da. The final list of identified proteins was generated by Scaffold 4.0.5 (Proteome Software, Portland, OR) with the following conditions: 99 % minimum protein probability, minimum number of 3 unique peptides, and 95 % peptide probability.

### HMT assay

Pax6 or control IgG antibody precipitates from nuclear extract as described above were incubated with 1 µg recombinant histones in the presence of [^3^H] S-adenosyl-l-methionine (SAM), for 1 h at 30 °C. The following procedure is done as we described earlier [80].

### Radiometric filter methyltransferase assay

N-terminal H3 peptides (1–20) containing unmodified K4, K4me1, K4me2, and K4me3 residues were obtained from Epicypher (catalog #: 12-0001, 12-0007, 12-0008, and 12-0009). The reactions (20 µl, final volume) were conducted with 1 µg of the peptide, specific immunoprecipitate (Pax6, Wdr5, and IgG control), [^3^H] SAM (0.55 µCi/µl, PerkinElmer), and BC200 buffer. After 1-h incubation at 30 °C, the reaction mixture was spotted on P81 phosphocellulose paper (Millipore) and washed 3× with sodium carbonate, pH 8.5 and 1× with acetone before air-drying. Four milliliters of scintillation cocktail was added to the filter paper, and emissions were counted. CPM were normalized to IgG control.

### Cell cultures and generation of shRNA cell lines

αTN4 cells are SV40 T-antigen-transformed mouse lens epithelial cells [81] that express many important lens-specific genes [82] and were maintained in DMEM F-12 with 10 % FBS. Lentiviral constructs expressing shRNAs including the controls were purchased from OpenBiosystems (Pax6 sh1: 5′-CCACTTCAACAGGACTCATTT-3′, Pax6 sh2: GCAAGAATACAGGTATGGTTT, and control sh: 5′-CTCGCTTGGGCGAGAGTAA-3′). Viral particles were produced by following the recommended protocols (Addgene). Two days after infection of cells with viruses, puromycin was added at 2 mg/ml to select for pooled populations of stably infected cells.

### ChIP-seq assays and peak calling

Ten 20-cm dishes of control and Pax6 sh2 αTN4 cells were cross-linked with 1 % formaldehyde at room temperature for 10 min and quenched by 2.5 M glycine. The ChIP was performed using antibodies as we described elsewhere [45]. Sequencing of Pax6 and histone ChIP-seq experiments was performed on Illumina HiSeq 2500 and Genome Analyzer IIx instruments. The ChIP-seq reads were analyzed by the Einstein WASP analysis pipeline [83] and aligned to the mouse genome (GRCm37/mm9) using Bowtie [84]. The data were deposited into GEO under accession numbers GSE66961 and GSE76315. For Pax6, RNA polymerase and histone modifications (H3K4me2 and H3K4me3) with sharp ChIP-seq profiles, peaks were called using the MACS2 program [85] using default settings. For histone modifications with broad ChIP-seq profiles (H3K4me1), peaks were called using the SICER program using default setting [86]. We filtered out peaks that mapped to the modENCODE blacklisted genomic regions [87]. The IGV Integrative Genomics Viewer (2.3.57) [88] was used for data visualization. Pax6 peaks overlapping at least 1 bp between lens and αTN4 cells are assigned as common peaks (*n* = 245) and others as specific peaks by BEDTools (v2.23.0) [89]. The peak overlap between the two cell types is significant (*p* < 2.2e-16 by Fisher’s exact test).

### Unbiased motif analysis at Pax6-bound regions

MEME (4.10.1) [90] and MEME-ChIP [91] were used to identify *de novo* enriched motifs with sequences −100 to +100 bp around Pax6 peak summits in lens and αTN4 cells; the setting was 6–20 bps as motif width and returning the top 10 motifs.

### Identification of Pax6 peaks association to genes and data visualization

The list of TSSs (transcription start sites) list was downloaded from the UCSC genome browser [92] using the RefSeq gene annotations [93]. Pax6 peaks overlapping at least 1 bp with the −2 to +2 kb around TSS were assigned as Pax6 peaks using the BEDTools (v2.23.0). The rest were considered as non-promoter peaks and further assigned to genes if they were within 50 kb of a gene as described previously [45]. The heatmaps to visualize histone modification were generated by Java Treeview [94] using the count matrix generated by the SeqMINER program [95], counting the read densities from −5 to +5 kb around Pax6 peak summits in 50 bp bins. During this analysis, the same numbers of ChIP-seq reads (~16 millions) were used, with a subset sampled from the full data set that had >16 million reads.

### qRT-PCR and RNA-seq experiments

Total RNA was extracted from αTN4 cells on a 6-well plate using TRIzol reagent and reverse-transcribed using the SuperScript III First-Strand Synthesis System (Invitrogen). Relative mRNA levels were normalized against average of Gapdh and B2 m. The library construction, sequencing, and data analysis were performed as we described previously [45]. The following primer pairs were used: B2m, 5′-CATACGCCTGCAGAGTTAAGC-3′, 5′-GATGCTTGATCACATGTCTCG-3′; Cap2, 5′-GGAAGCAACATGTTCAACCA-3′, 5′-CGTCGTTCATCTCCTTGACA-3′; Farp1, 5′-CCAGGGAAGGTTCTGTTTGA-3′, 5′-ACCACGATCTTCCTGTGGTC-3′; Gapdh, 5′-CTTCCGTGTTCCTACCC-3′, 5′-TGCTGTAGCCGTATTCAT-3′; Pax6, 5′-GCACATGCAAACACACATGA-3′, 5′-ACTTGGACGGGAACTGACAC-3′; Plekha1, 5′-GACAGAATCGCATCTGTGGA-3′, 5′-TGAAGGCAGGTTCTGTGGAT-3′; Prox1, 5′-TGACTCGGGACACAACAAGT-3′, 5′-ATCTCTCTGGAACTGCGCTT-3′; Tshz2, 5′-GCGGCAAGAATGATTTTGAT-3′, 5′-ATAGCTGCACGGAGCTGAAT-3′; and Zfp536, 5′-CAATGGGCAGAACCTAGGAA-3′, 5′-ATGCTATTGAACCGGAAACG-3′.

### Analysis of conditionally inactivated Pax6 in RPE cells

The mouse lines employed in this study, Pax6^loxP^ [31] and DctCre [96], have been previously described. The genetic background of all mice used in this study was C57BL/6J. All animal work was conducted according to national and international guidelines and approved by the Tel Aviv University Review Board. For RNA isolation, RPEs of control *Pax6*^*loxP/loxP*^ and mutated *Pax6*^*loxP/loxP*^; DctCre mice were dissected at E15.5 and RNA was extracted using QIAshredder and RNeasy kits (Qiagen). Reverse transcription of 1 µg of RNA from each sample was performed using qScript cDNA Synthesis Kit (Quanta). cDNA was amplified using the Power SYBR Green Mix (Applied Biosystems) in a 384-well optical reaction plate using ABI Prism 7000 Sequence Detection System (Applied Biosystems). Plekha1 primers used for qRT-PCR are described above, and relative expression was normalized using Tbp and Hprt transcripts. Immunofluorescence analysis was performed on 10-µm paraffin sections as previously described [96] using primary rabbit anti-Plekha1 antibody and secondary antibody of donkey anti-rabbit conjugated to Alexa 594 (1:1000, Invitrogen, A21207).

## Additional files

10.1186/s13072-016-0087-z Complete list of Pax6 associated proteins identified by MS
10.1186/s13072-016-0087-z Semi-quantitative analysis of Ash2 l and Rbbp5 expression and global histone methylation in Pax6 KD cells. **a** Western immunoblotting to show that Pax6 KD does not impair expression levels of common subunits, Ash2 l and Rbbp5. Expression of basal transcription factor TBP was used as loading control. **b** Western immunoblotting to show that Pax6 KD does not affect methylation levels of histone H3, including H3K4m1, H3K4me2, H3K4me3, H3K79me2 and H3K9me2. Fig. S2. Transcription factor binding sites in the evolutionarily conserved distal region of Plekha1. Two Pax6 binding sites were identified within the Pax6 ChIP-seq peak (Fig. 8a, region A). In addition, analysis of surrounding sequences predicts two Sox- and one large Maf-binding sites. The Sox- and Maf-binding motifs are from JASPAR database (Sox motif ID: 15863505; Maf motif ID: 9571165). The Pax6 motif is based on ChIP-seq studies in αTN4 cells (see Fig. 4b).

## Abbreviations

ChIP: chromatin immunoprecipitation
qChIP: ChIP-qPCR
co-IP: co-immunoprecipitation
HAT: histone acetyltransferase
HMT: histone methyltransferase
KD: knockdown
LC–MS/MS: liquid chromatography-tandem mass spectrometry
PTM: posttranslational modification
RPE: retinal pigment epithelium
WT: wild type
