# Supplementary material for: Pax6 associates with H3K4-specific histone methyltransferases Mll1, Mll2, and Set1a and regulates H3K4 methylation at promoters and enhancers
Source: Epigenetics Chromatin. 2016 Sep 9;9(1):37. doi: 10.1186/s13072-016-0087-z (PMC5018195; doi:10.1186/s13072-016-0087-z)
Supplement: Supplementary file 2 — 10.1186/s13072-016-0087-z Semi-quantitative analysis of Ash2 l and Rbbp5 expression and global histone methylation in Pax6 KD cells. a Western immunoblotting to show that Pax6 KD does not impair expression levels of common subunits, Ash2 l and Rbbp5. Expression of basal transcription factor TBP was used as loading control. b Western immunoblotting to show that Pax6 KD does not affect methylation levels of histone H3, including H3K4m1, H3K4me2, H3K4me3, H3K79me2 and H3K9me2. Fig. S2. Transcription factor binding sites in the evolutionarily conserved distal region of Plekha1. Two Pax6 binding sites were identified within the Pax6 ChIP-seq peak (Fig. 8a, region A). In addition, analysis of surrounding sequences predicts two Sox- and one large Maf-binding sites. The Sox- and Maf-binding motifs are from JASPAR database (Sox motif ID: 15863505; Maf motif ID: 9571165). The Pax6 motif is based on ChIP-seq studies in αTN4 cells (see Fig. 4b). [file 13072_2016_87_MOESM2_ESM.pdf]

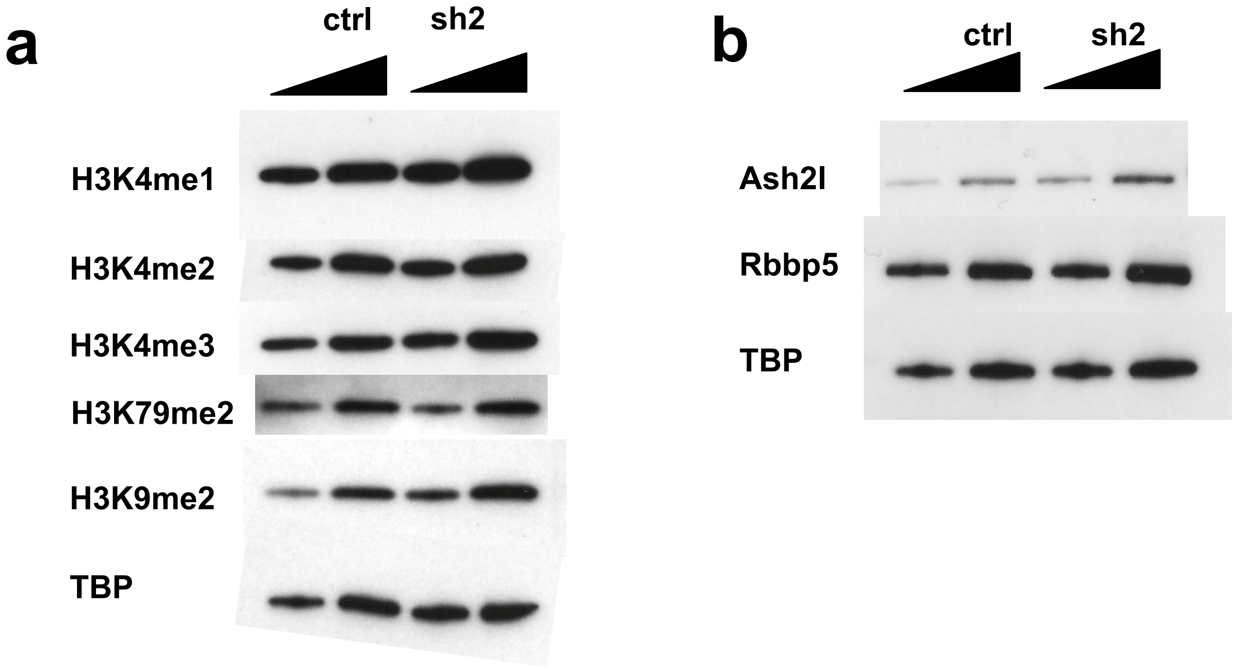

a
ctrl
sh2
H3K4me1
H3K4me2
H3K4me3
H3K79me2
H3K9me2
TBP
b
ctrl
sh2
Ash2l
Rbbp5
TBP

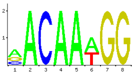

```
                                                    Sox2
Mouse     ------------------------------------------TCACGCACAAAGG---TC
Rat       ------------------------------------------CCACGCACAAAGG---TC
Rhesus    ------------------------------------------CAGCACTCATATC---TC
Horse     TTGCGCTTTCTGTTCCTCCACCTGGAACTGTCCTCCCTACATCCTCACAGAGCTGGCCTC
Dog       TTGTACGTTCTGTTCCTCTGCCTGGAACAGTCTTCCC-AGATCCTCACACAGATGGCCTC
Human     ------------------------------------------CAGCACTCATATC---TC
                                                    .  *.*  *       **
```

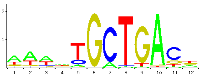

```
            Maf
Mouse     GCCTAATTAATTAGCTGACTCCCCCCAGCGTAATGACTGAGGACGGAGGCTGGAGGTCTG
Rat       GCCTAATTAATTAGCTGACT-CCCCCGGCGTAATGACTGAGGATGGAGGATGGAGGTCTG
Rhesus    AGTGAATTAATTAGTCGACG-CCCCAAGCATA----CTGAGGGTGGAGGGCGGGTGCCTG
Horse     TGCTTATCAGTCAGGTCTCA-GCTCCAGGTCACCCCCTCA-GGTGGCTTCCCTGAACCC-
Dog       TGCTTATCACTCCGGTCTCA-GCTCCCACTCACCCCCTTGGGGTGGCTTCCCCCAACCCG
Human     AGTGAATTAATTAGTTGACG-CCCCAAGCATA----CTGAGGGTGGAGGGCGGATGCCTG
            .  **.* *. * .  *   *.*  .  .*    ** . *..**    .    ..*.

Mouse     GGATCTGAGAGCCACATG----------------------CAGCCTATCTCTCAAGTCCA
Rat       GGATCTGAAAGCCACATG----------------------CAGCCTATCTCTC-AGTCCA
Rhesus    GGACTTGAAAG--ACATG----------------------TGGCACATCTCCA-GGTCCA
Horse     ---TCCAGGTGCTCCCTGCCTCATCAGTCTATTTTCTTCCTGCTGCACATCTA-AGCCCC
Dog       ---TCCAGGTGCTCTCTGCTGCA------TATTTTCTTCCTGCCGTGCACCTA-AGTTCA
Human     GGACTTGAAAG--ACGTG----------------------TGGCACATCTCCA-GGTCCA
             ...... *   . **                      .. . ... .*.  .*..*
```

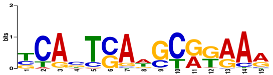

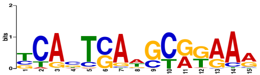

```
        Pax6                    Pax6
Mouse     CGGCTCACTGAGCTGGAAAATCTCCCAAACCAGCCAAGTGGAAGGGGCACAGAAGG-AGA
Rat       CGGCTCACTGAGCTGGAAAATCTCCCAAAGCAGCCAAGTGGAAGGGGCACAGAAGG-AAA
Rhesus    CTGTGCGCTGAGCTGGAAGATTTCAGAAATGAACCATGTGGAAACAGCACAGGAAGCCCA
Horse     CTGCTCACTGGGCTGGAAAATCTCAGCAACCAGACAAGTAGAAAAGGTGCAGGAAGCCCA
Dog       CTGCTCACTGGTCTGGAAAATCTCACAAGCTAGCCAAGT-GAAAAGGCACAGGAAGCCCA
Human     CTGCGCACTGGGCTGGAAGATTTCAGAAACGAGCTATGTGGAAACGGCACAGGAAGCCCA
          * *. *.***. ******.**.**   *.  *. .* ** ***. .*..***.*.*   *
```

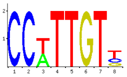

```
            Sox2
Mouse     TAAAGGAATTTTTCCATTGTGGTGGCCTGAGGGGG--TC
Rat       TAAAGGGACTTTTCCATTGTGGTGGCCTGAGGGGG--TC
Rhesus    CAGAGCGATTCTTCCATTGCAGTGGGCTGAGGGAGGGTC
Horse     CGAAGTGATT-TTCCTCTGTAGTGGCCTGAGCGAGGGTC
Dog       TGAAGTGATT-TTCCATCAGGATGGCCTGAGTGAGGGTC
Human     CAGAGCGATTCTTCCGTTGCAGTGGCCTGAGGGAGGGTC
          ...** .*.* **** ... ..*** ***** *.*  **
```
